# Diaphyseal giant cell tumor with multiple relapses in a skeletally immature patient: a case report

**DOI:** 10.11604/pamj.2022.41.13.27763

**Published:** 2022-01-06

**Authors:** Ibrahim El Shamly, Olivier Kubwimana, Thomas Habanabakize, Muvunyi Jean Baptiste, Thierry Zawadi Muvunyi, Marie Grace Kansayisa

**Affiliations:** 1 Center for Preservation and Transplantation of Musculoskeletal Tissues, Cairo University, Cairo, Egypt,; 2 Department of Surgery, University of Rwanda, Kigali, Rwanda,; 3 Department of Pathology, University of Rwanda, Kigali, Rwanda,; 4 Department of Pathology, King Faisal Hospital, Kigali, Rwanda,; 5 Kigali University Teaching Hospital, Kigali, Rwanda

**Keywords:** Diaphysis, giant cell tumor, skeletal immaturity, osteolytic, case report

## Abstract

Giant cell tumor (GCT) is an aggressive osteolytic lesion mostly affecting the meta-epiphyses of long bones at skeletal maturity. Occurrence of the GCT in diaphysis is a rare entity in adult and exceptionally rare in pediatric population. This is the only third diaphyseal case reported in pediatric population. We report a case of recurrent diaphyseal GCT in a skeletally immature patient of 15-year-old male at the right radius after previous resection with plate and screw fixation. Upon optimal investigations, en-bloc resection of the tumor with radial resection and ulna centralization with wrist arthrodesis was done for a campanacci stage III GCT. The patient had an uneventful recovery without recurrence for 2 years and 2 months following surgery. The main challenge relies on accurate diagnosis due to uncommon location that hinders adequate treatment plan, therefore diagnosis should be solely based on histopathology findings.

## Introduction

Giant cell tumor (GCT) of the bone is a benign tumor locally aggressive with osteolytic lesions affecting the meta-epiphysis at skeletal maturity, typically in the second to fourth decade of life [1,2]. GCT accounts 5-6% of primary bone tumors [3]. Common tumor location are distal femur and proximal tibia [2]. Around 85% of GCT cases occur in the meta-epiphyseal area with a female gender preponderance [4]. Diaphyseal true GCT is a very rare entity and accounts for an average 2% and may mimic other tumors [5]. The incidence of 1.8-10.6% was reported as pediatric bone GCT [6,7]. GCT typically present with pain, swelling, progressive non-weight bearing with 10 to 30 % of pathologic fractures at presentation [1,3]. Standard radiography, magnetic resonance imaging (MRI) and biopsy make the hallmark of investigations [1,8]. Histology reveals bone erosion surrounded with multinucleated giant cells which is pathognomonic. Those giant cells result from fusion of multiple recruited monocyte when stimulated by receptor activator of nuclear factor kappa-B ligand (RANKL) and upon their formation GCT presents receptor activator of nuclear factor kappa-B (RANK) receptors where RANKL will further bind, inducing multinucleated giant cells signaling to absorb the bone hence osteolysis [2].

The standard management options include appropriate curettage and use of bone cement, with local or systemic adjuvant therapy as adjunct [1,2]. Recurrences are treated by extended wide excision with bone graft [9]. Denosumab is a IgG2 human monoclonal antibodies product capable of abolishing multinucleated giant cells activity [2,10]. Despite optimal surgical management the recurrence rate is 30-50% [1]. The relatively uncommon occurrence of GCT heavily impact on the feasibility of clinical trials to propose adequate management protocols, witnessed by the fact that there have not yet a significant advance in treatment options for the past three decades hence scattered GCT management protocols. There are scarce data in our region about musculoskeletal tumors and there have not been any previously reported case or study about GCT in sub-Saharan Africa.

## Patient and observation

**Figure 1:**
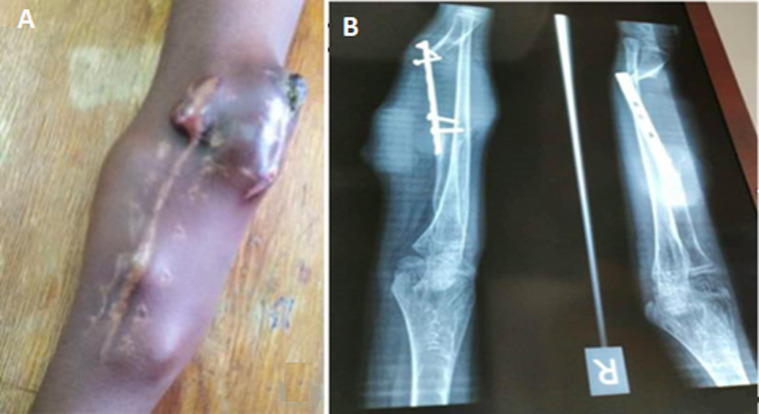
15-year-old boy with diaphyseal giant cell tumor: A) multiple masses on right forearm with the most prominent at the proximal aspect of the antecubital fossa; B) radiograph of the right forearm; although image of poor quality, a relatively intact epi-metaphysis of the distal radius can be noticed; the soft tissue shadowing on a previously resected diaphysis with lytic like features and distal screws pullout are identified

**Clinical findings and timeline of current episode:** a 15 year-old-male patient from a low socio-economic background presented to our tertiary hospital with right forearm multiple recurrent masses for the past 6 months. The child originates from the Southern Province of Rwanda and has been consulting different public health facilities and traditional healers without significant outcome. Upon consultation, he was complaining of multiple right forearm, moderately painful swellings with progressive increase in size. Fifteen months´ prior consultation he underwent tumor excision together with open reduction and internal fixation (ORIF) with plate and screws at a teaching hospital in his province of origin, after a tissue biopsy had confirmed the tumor as GCT. The surgery was done by an expatriate surgeon from a missionary campaign. Later after 3 months the tumor progressively relapsed and the parents re-consulted and were transferred to our tertiary hospital. His initial exhaustive clinical examination was done and revealed multiple right forearm swelling closer to previous right radial and volar incision (Figure 1 A). The swellings were soft, non-tender with fluctuation.

**Figure 2:**
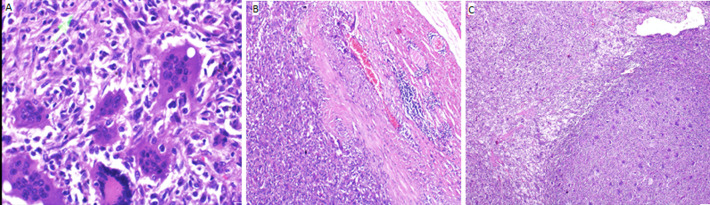
(A,B,C) photomicrographs with hematoxylin and eosin stain showing; A) dermal cell well circumscribed mesenchymal neoplasm composed of numerous osteoclast-like and touton giant cells evenly distributed in cellular stroma of oval to spindle cells (marked green arrow C); B) there is mild atypia and some mitotic figures; C) no atypical mitoses observed

**Diagnostic assessment:** baseline pre-operative work up including parathyroid hormone were done and revealed normal values. A pre-operative radiograph (Figure 1 B) was done and printed in another hospital and could not be retrieved at the time of publication as the patient had lost the radiograph printout. However, we can notice the sparing of epi-metaphysis of distal humerus with hardware in-situ. Initial working diagnosis was recurrent simple bone cyst, aneurysmal bone cyst, chondromyxoid fibroma and giant cell sarcoma. Biopsy taken and staining revealed lobulated giant cell with mono-nuclear appearance of mesenchymal cells (Figure 2). The computed tomography (CT) scan of the chest did not show lung metastasis and MRI could not be safely performed due to the fact that our patient had an unknown implant type used at the primary surgery.

**Figure 3:**
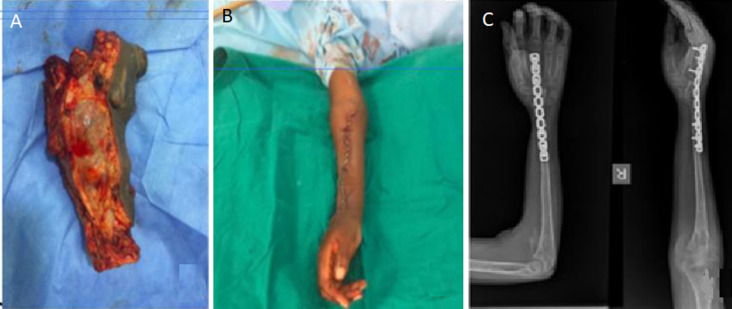
post en bloc resection and wrist fusion: A) macroscopic appearance of the resected mass with fluid-fluid like lobules; B) post-operative wound whereby primary wound closure was achieved; previous scars with relapses were included in the en bloc tumor resection to ensure adequate surgical margins; C) radiography of the right forearm with centralized ulnar

**Therapeutic interventions:** after adequate optimization, he was operated on 4^th^ August 2019 with near-total radial excision with both two previous scars included in the incision to achieve a safety margin and the ulna was centralized (Figure 3 A,B). A wrist arthrodesis with a ten hole-reconstruction (Figure 3 C) plate was done and the wound was primarily closed with an above elbow back slab applied. The resected specimen encased macroscopically previous stainless steel contact dynamic compression (DCP) plate with pulled screws. Post-operative tissue analysis kept the diagnosis as GCT with achieved safe margins. Patient had an uneventful recovery and discharged on third post-operative day.

**Follow-up and outcome of interventions:** two weeks later the wound was healed and sutures were removed and the slab was converted into complete cast for three weeks to allow consolidation of the arthrodesis. Two years and 2 months post-surgery our patient is doing well; pain free without relapse and has returned to school. He has grip strength of 60% compared to the normal contralateral side, without a thumb extension lag and n musculoskeletal tumor society (MSTS) score of 27.

**Informed consent:** we appreciate the parents of our patient who willingly gave us their permission and consent to publish this report.

## Discussion

Apropos of pediatric group, diaphyseal giant cell tumor has only been described twice in the literature and one of them was made by Patel *et al*. [8] and probably our case is the third to be reported and the ninth case overall when encasing the adult population case reports [5] and the entire summary is available in Table 1. All cases had no underlying previous medical illness and had good outcome except one recurrence after curettage. A distinct pure diaphyseal GCT is an unprecedented finding before growth plate closure [1,8]. The current case shared similarities with variously reported cases in regard to the symptoms progression, tumor grading and management.

**Table 1:** details of the 9 case reports of diaphyseal giant cell tumor

Author	Geographic region	Age and sex	Anatomical location	Biopsy (+/-) and type (FNA, incisional)	Procedure done	Associated factors	Outcome	Follow-up period
**Skeletally immature**								
Patel *et al*. 2015	India	15 years female	Diaphysis ulnar	+/incisional	Resection + bone graft	None	Good	2 years
Visscher *et al*. 1988	US	7 months male	Diaphysis ulnar	+/not specified	En-bloc resection and fibular graft	None	Good	1 year 3 months
Our current case	Rwanda	15 years male	Diaphysis radius with multiple relapses	+/incisional	En-bloc resection and ulnar centralization	None	Good	2 years 2 months
**Skeletally mature**								
Sandeep *et al*. 2008	India	35 years female	Diaphysis radius	+/FNA	Resection and centralization of ulnar	None	Good	2 years
Fain *et al*. 1993	US	21 years female	Diaphysis tibia	+/not specified	Curettage and bone graft	None	Recurrence after 6 years	4 years
		27 years female	Diaphysis tibia	+/not specified	Curettage	None		26 years
		37 years female	Meta-diaphysis fibula	+/not specified	En-bloc resection	Noe		5 years
Darioush *et al*. 2013	Iran	46 years female	Meta-diaphysis femur	+/incisional biopsy	Curettage and bone cement	None	Good	2 years
Wilkerson *et al*. 1969	US	27 years female	Diaphysis tibia	-		None	-	-

FNA: fine needle aspiration; -: no details found

Progressive pattern of pain with quite soft swelling has been similarly described in literature and the tumor fit the campanacci type III description referring to tumor invasion, cortical break through and soft tissues involvement [1,2]. The original radiological evidences of our case were not obvious as the presented imagings were done after the initial surgery however multiply relapsed and lytic lesions involving the diaphysis of the radius with soft tissue shadows could be observed. Although the MRI couldn´t be requested due to hardware in-situ, the histopathological findings of the specimen revealed a benign neoplasm composed of mononuclear round to spindle cells with numerous evenly dispersed osteoclast-like giant cells without evidence of malignant transformation; features consistent with GCT, similar to the previous documentation in the literature [5].

The present case was a relapsed bone GCT which correlates with the available literature findings reporting the incidence of recurrence amongst pediatric age group to be around 20% as reported by Puri *et al*. [7]. The former management of GCT by performing curettage with or without bone grafting has shown a high recurrence that led to further treating options [11]. Interestingly, Li *et al*. study found copious curettage while avoiding soft tissue contamination was a sufficient measure to prevent recurrence compared to inter-lesional curettage, with recurrence rate of 41.9% and 19% respectively [12]. Advances were therefore set in such as bone cement through its exothermic reaction, high speed burr, adjuvant treatment, radiotherapy. Minimally invasive approaches with computer assisted technique like cryosurgery are on the rise with low recurrence [13]. Use of high speed burr allow a wider curettage and decrease recurrence risk [12], albeit recent animal study depicted a possibility of tumor seeding [14]. Recurrence in GCT can be challenging and requires a more aggressive approach like en-bloc excision especially after, as similarly done in our case [6]. Our aforementioned procedure technique was found effective by Meena *et al*. although limited hand function [9].

## Conclusion

Epi-metaphyseal GCT in adults is aggressive in nature with high recurrence rate. Sporadic incidence of diaphyseal GCT in skeletally immature population group can potentially alter early adequate treatment plan. Appropriate tissue biopsy is therefore primordial. Our case presented noteworthy traits: tumor appearance at such a tender age and diaphyseal location with multiple relapses. Long term evaluation of our patient will allow us to have a timely clinical assessment to depict any potential recurrence.

## References

[1] Mavrogenis AF, Igoumenou VG, Megaloikonomos PD, Panagopoulos GN, Papagelopoulos PJ, Soucacos PN (2017). Giant cell tumor of bone revisited. Sicot-J.

[2] Singh AS, Chawla NS, Chawla SP (2015). Giant-cell tumor of bone: treatment options and role of denosumab. Biologics.

[3] Zanati A, Ferreira N, Marais L (2016). Giant cell tumour of bone: a demographic study from a tumour unit in South Africa. SA Orthop J.

[4] van der Heijden L, Dijkstra PD, van de Sande MA, Kroep JR, Nout RA, van Rijswijk CS (2014). The clinical approach toward giant cell tumor of bone. Oncologist.

[5] Fain JS, Unni KK, Beabout JW, Rock MG (1993). Nonepiphyseal giant cell tumor of the long bones Clinical radiologic, and pathologic study. Cancer.

[6] Campanacci M, Baldini N, Boriani S, Sudanese A (1987). Giant-cell tumor of bone. J Bone Joint Surg Am.

[7] Puri A, Agarwal MG, Shah M, Jambhekar NA, Anchan C, Behle S (2007). Giant cell tumor of bone in children and adolescents. J Pediatr Orthop.

[8] Patel MT, Nayak MR (2015). Unusual presentation of giant cell tumor in skeletally immature patient in diaphysis of ulna. J Orthop Case Rep.

[9] Meena DK, Thalanki S, Sharma SB (2016). Wrist fusion through centralisation of the ulna for recurrent giant cell tumour of the distal radius. J Orthop Surg (Hong Kong).

[10] Lipplaa A, Dijkstra S, Gelderblom H (2019). Challenges of denosumab in giant cell tumor of bone, and other giant cell-rich tumors of bone. Curr Opin Oncol.

[11] Salunke AA, Shah J, Warikoo V, Chakraborty A, Pokharkar H, Chen Y (2017). Giant cell tumor of distal radius treated with ulnar translocation and wrist arthrodesis: what are the functional outcomes. J Orthop Surg (Hong Kong).

[12] Li D, Zhang J, Li Y, Xia J, Yang Y, Ren M (2016). Surgery methods and soft tissue extension are the potential risk factors of local recurrence in giant cell tumor of bone. World J Surg Oncol.

[13] Deslivia MF, Savio SD, Dharmapradita MW, Wiratnaya IGE (2019). Efficacy of minimally invasive surgery on giant cell tumour of the bone: a systematic review. Open Access Maced J Med Sci.

[14] Wang PH, Wu CL, Chen CM, Wang JY, Wu PK, Chen WM (2020). Adjuvant therapy by high-speed burr may cause intraoperative bone tumor seeding: an animal study. BMC Musculoskelet Disord.

